# A new species of *Cyanea* (Campanulaceae, Lobelioideae) from Maui, Hawaiian Islands

**DOI:** 10.3897/phytokeys.13.3447

**Published:** 2012-06-20

**Authors:** Hank Oppenheimer, David H. Lorence

**Affiliations:** 1 Plant Extinction Prevention Program, Pacific Cooperative Studies Unit, University of Hawaii, P.O Box 909, Makawao, HI 96768, USA; 2 National Tropical Botanical Garden, 3530 Papalina Road, Kalaheo, HI 96741, USA

**Keywords:** Campanulaceae, conservation, *Cyanea*, Hawaiian Islands, IUCN Red List, Maui

## Abstract

*Cyanea kauaulaensis* H. Oppenheimer & Lorence, **sp. nov.**, a new, narrowly endemic species from Maui, Hawaiian Islands is described, illustrated with field photos, and its affinities and conservation status are discussed. It is currently known from 62 mature plants and is restricted to Kaua`ula and Waikapu valleys on leeward western Maui. It differs from all other species of *Cyanea* by its combination of many-branched habit; glabrous, unarmed, undivided leaves; small, narrow, glabrous corollas with small calyx lobes that do not persist in fruit; and bright orange, subglobose to obovoid fruits.

## Introduction

**Figure 1.**
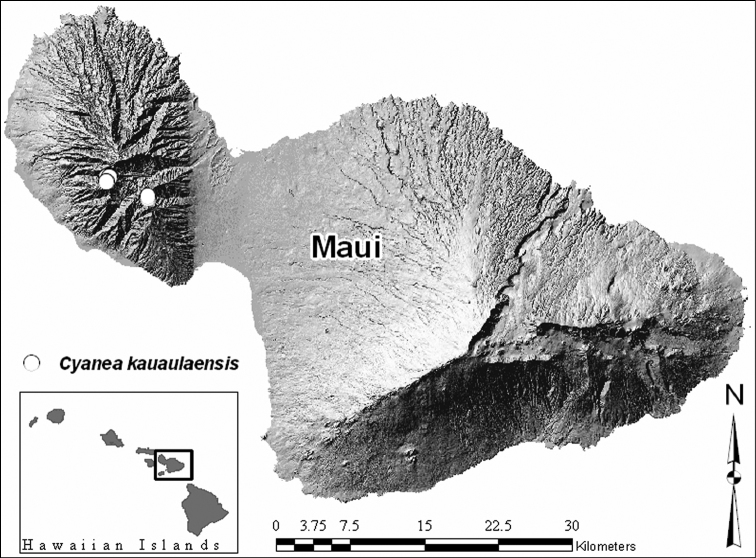
Distribution map of *Cyanea kauaulaensis* showing known localities on East Maui.

As currently circumscribed, the woody lobelioid genus *Cyanea* Gaudich. (including *Rollandia* Gaudich.) comprises 78 species (Lammers 2007), all endemic to the Hawaiian Islands where they occur in wet and mesic forests. The Hawaiian lobeliads are the largest plant clade restricted to any archipelago, with *Cyanea* being the largest genus within that clade. It is also the largest genus in Hawai`i, and originated 8-10 Mya (Givnish et al. 2008). *Cyanea* was first described by Gaudichaud-Beaupré (1824) based on the type species *Cyanea grimesiana* Gaudich. The genus was later treated in Rock’s (1919) monographic study of the Hawaiian Lobelioideae in which he recognized 52 species in 5 sections. Wimmer (1943) recognized only 3 sections in his monograph of Campanulaceae. Lammers (1990) revised the Hawaiian members and also recognized 52 species but stated relationships within *Cyanea* remained poorly understood and consequently did not recognize any formal sections. Recent exploration and collecting efforts in poorly explored, often rugged or remote regions in the Hawaiian Islands continue to yield undescribed species of *Cyanea* (Lammers 2004, Lammers and Lorence 1993). In 1989, during a botanical survey of lands then owned by Pioneer Mill Co. on West Maui (currently Makila Land Co.), about a dozen plants of an unusual Campanulaceae were found along the southern fork of the amphitheatre headwaters of Kaua`ula Valley (Fig. 1). The botanists on that excursion, Steve Perlman, Sam Gon, and Robert Hobdy, easily recognized the plants as belonging to the endemic Hawaiian genus *Cyanea*. However, these plants were misidentified and thought to represent *Cyanea glabra* (F. Wimmer) St. John [based on *Cyanea knudsenii* Rock var. *glabra* F. Wimmer], a species previously known to be endemic to wet forest of windward East Maui and possibly extinct. Recent research in the north fork of Kaua`ula Valley during 2008 and 2009 resulted in the discovery of additional plants. In July of 2011 a new population was discovered in Waikapu Valley nearly 5 km from the previously known plants and separated by several large valleys and canyon walls and ridges 700 m in height.

Critical study of these collections reveals that they are not *Cyanea glabra* nor even closely related to it, and clearly represent an undescribed species. Based on the description in Lammers (1990) and examination of type collections at the BISH and W herbaria, *Cyanea glabra* differs from this new species in having dimorphic leaves which are pinnatifid in juvenile plants with aculeate petioles and both surfaces of the midrib and veins, adult plants with 6–8-flowered inflorescences, flowers with larger hypanthium 7–10 mm long, larger calyx lobes 2–8 mm long, larger, often purple-tinged, strongly curved corollas 50–60 mm long, and somewhat larger, ellipsoid fruits 10–15 mm long crowned by the persistent calyx lobes. For consistency with other treatments and descriptions, measurements given below are based on dried herbarium specimens, although descriptions have been supplemented with pickled material and field photos.

## Systematics

### Cyanea kauaulaensis

H. Oppenheimer & Lorence sp. nov.

urn:lsid:ipni.org:names:77120339-1

http://species-id.net/wiki/Cyanea_kauaulaensis

Figures 2 , 3

#### Latin.

*Species allied to Cyanea profuga C. Forbes, differs in its narrower leaves 5–7 cm wide, inflorescences with more numerous flowers (up to 20); flowers with smaller, lanceolate to linear calyx lobes 2–3 × 0.5–0.7mm, apex acute to acuminate, shorter than the hypanthium, comparatively shorter corolla lobes 1/3-2/5 as long as the tube, and subglobose to obovoid orange fruits*.

#### Type.

**USA.** **HAWAIIAN ISLANDS:** West Maui: Lahaina District, N fork of Kaua`ula Valley, 910m (3000 ft), 2 Dec 2008, H. Oppenheimer & S. Perlman H120806(Holotype PTBG-058138 [+ spirit collection]!; Isotypes BISH!, US!).

#### Description.

*Unarmed shrubs* 2–4 m high, many-branched from the base with many basal shoots, stems light brown, erect to arching, up to 6m long, sometimes leaning on adjacent vegetation, often rooting where in contact with soil, leaf scars subcircular to broadly obovate-depressed; latex white. *Leaves* clustered near the end of the branches, when fresh light green on both surfaces, fleshy, the adaxial surface moderately glossy, drying membranaceous to chartaceous, glabrous on both surfaces, in juvenile plants occasionally minutely muricate adaxially along costa, elliptic, oblong, or elliptic-oblong, in adult plants blade 19–30 × 5–7 cm, base attenuate to cuneate, often asymmetrical, apex attenuate, acuminate, or cuspidate, margins entire to minutely serrulate-dentate, often undulate when fresh, sometimes coarsely serrate-dentate in juvenile plants; petioles 5–10 cm long, glabrous. *Inflorescences* axillary and on leafless nodes, developing along stems after leaves have fallen on well developed individuals, up to 30 per stem, mostly perpendicular to the stem, 5–20 flowered, peduncles 15–70 mm long, glabrous. *Flowers* on pedicels (4) 8–12 mm long, filiform, glabrous, subtended by caducous linear-subulate to linear bracts 12–20 × 3 mm, margins minutely serrulate, glabrous, pedicels with 2–3 glabrous subulate-oblong bracteoles 0.3–0.6 mm long; hypanthium 4–5 × 2.5–4 mm, broadly ellipsoid to obovoid-obconic; calyx lobes 2–3 × 0.5–0.7 mm, lanceolate to linear, apex acute to acuminate, caducous in fruit; corolla white, tubular, round in cross section, gently curved to suberect, 28–35 × 3–4 mm, externally glabrous, internally minutely papillose, the tube 23–27 mm long, the lobes 5–10 mm × 0.5–0.9 mm medially, linear-subulate, reflexed, initially 1/3 to 2/5 as long as the tube but eventually splitting more deeply; staminal column glabrous, anthers 6–7 mm long, glabrous, the lower 2 with apical tufts of white hairs 3–4 mm long. *Fruits* bright orange when ripe, 8–10 mm in diameter, globose to obovoid, smooth, apex crowned by an apicular ring, calyx lobes caducous very early when fruits still small and green; old infructescences often producing leaves and continuing to develop as lateral shoots. *Seeds* numerous, embedded in translucent pulp, ovoid-ellipsoid, 0.5–0.6 × 0.35–0.4 mm, testa brown, shiny, smooth with faint striations.

#### Distribution.

Known only from West Maui, Hawaiian Islands.

#### Habitat and ecology.

**Figure 2.**
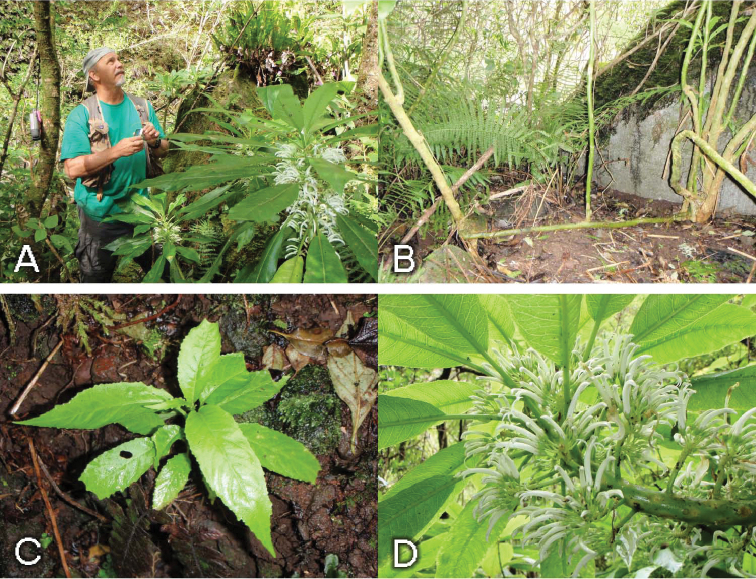
*Cyanea kauaulaensis*. **A** habit, with Steve Perlman (*Oppenheimer & Perlman H120806*) **B**  base of plants showing new stems arising from decumbent branch (*Oppenheimer & Perlman H120806*) **C**  juvenile plant (*Oppenheimer & Wood H20928*) **D** flowering stem (*Oppenheimer & Perlman H120806*); photos by H. Oppenheimer.

**Figure 3.**
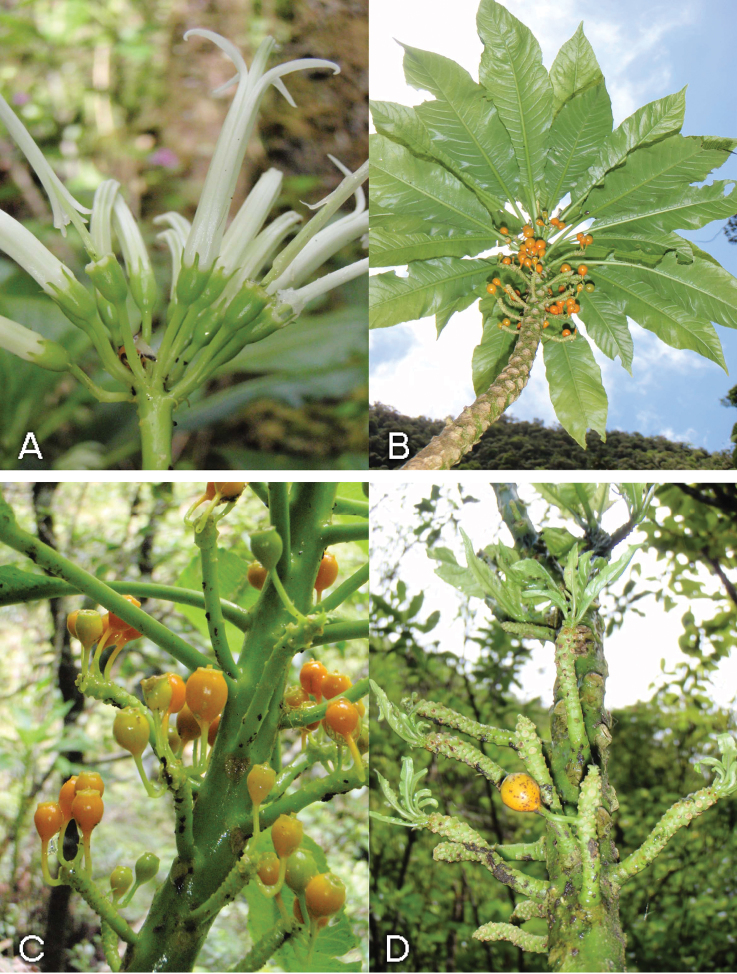
*Cyanea kauaulaensis*. **A** inflorescence (*Oppenheimer & Perlman H120806*) **B** fruiting stem (*Oppenheimer et al. H40919*) **C** infructescences (*Oppenheimer et al. H40919*) **D** old infructescences becoming lateral shoots (*Oppenheimer & Wood H20928)*; photos by H. Oppenheimer.

*Cyanea kauaulaensis* occurs in riparian sites, on talus or basalt boulder-strewn slopes along perennial streams at elevations of 732 to 914 m. The plant community represents a *Metrosideros* Banks ex Gaertn. lowland wet forest. The most common associated woody elements are species of *Antidesma* L., *Boehmeria* Jacq., *Broussaisia* Gaud., *Cheirodendron* Nutt. ex Seem., *Clermontia* Gaud., *Coprosma* J.R. Forst. & G. Forst., *Cyrtandra* J.R. Forst. & G. Forst., *Dodonaea* Mill., *Dubautia* Gaud., *Ilex* L., *Kadua* Cham. & Schltdl., *Perrottetia* Kunth, *Pipturus* Wedd., *Psychotria* L., *Urera* Gaud., and *Xylosma* G. Forst. Ferns including species of *Asplenium* L., *Cibotium* Kaulf., *Cyclosorus* Link, *Deparia* Hook. & Grev., *Diplazium* Sw., *Dryopteris* Adans., *Elaphoglossum* Schott ex J. Sm., *Microlepia* C. Presl, *Pteris* L., *Sadleria* Kaulf., *Tectaria* Cav., and *Vandenboschia* Copel. are prevalent. *Freycinetia arborea* Gaud. is a widespread liana, and several herbaceous species of *Peperomia* Ruiz & Pav. are also present. The sedge genera *Machaerina* Vahl. and *Rhynchospora* Vahl are also frequent. Soil is of typical basaltic origin. The average annual rainfall is approximately 3000 mm. Due to the steep canyon walls, often 700 m tall, direct sunlight is restricted to midday, and varies seasonally. Plants occur on both sides of the streams, with no apparent preference. Adult plants are clumped (Fig. 2A) and often many branched from the base, the decumbent branches often rooting when in contact with the ground and forming “runners”, often with erect shoots (Fig. 2B). Stems are erect to ascending or vine-like, to 6m long, often leaning on and tangled with adjacent vegetation, growing on lower talus slopes in riparian areas along perennial streams. On some stems old infructescences were observed to produce leaves and continue to grow as lateral shoots (Fig. 3D). This may represent a mechanism for producing lateral branches.

#### Phenology.

*Cyanea kauaulaensis* has been observed flowering from late summer through January, followed by fruits maturing in March and April. Sporadically, some individuals may possess a few flowers or fruits earlier in summer.

#### Etymology.

The specific name honors Kaua`ula Valley, a large, amphitheatre-headed valley on leeward Mauna Kahalawai (aka West Maui). *Lit*., the red rain (Pukui et al. 1966), + Latin suffix *-ensis*, indicating a place of origin or growth. Kaua`ula Valley is an important site not only botanically, but economically (as a water source) as well as culturally and spiritually for Native Hawaiians.

#### Conservation status.

*Cyanea kauaulaensis* should be considered Critically Endangered due to its limited range, low population numbers, lack of population structure and poor seedling recruitment, probable loss of most or all of its avian pollinators and dispersal agents, landslides, flooding, herbivory by alien slugs and rats, and competition with alien plants such as *Ageratina adenophora* (Sprengel) R.M. King & H. Rob.,* Buddleia asiatica* Lour.,* Coffea arabica* L.,* Cortaderia jubata* (Lemoine ex Carrière) Stapf,* Erigeron karvinskianus* DC,* Macaranga tanarius* (L.) Müll. Arg., *Melinis minutiflora* P. Beauv.,* Rubus rosifolius* Smith,* Setaria palmifolia* (J. König) Stapf. and *Toona ciliata* M. Roem. Approximately 45 plants plus four seedlings were observed during the recent visits in Kaua`ula Valleys north fork, three in the south fork, and 12 in Waikapu Valley. Recently, a new species of the endemic Hawaiian genus *Stenogyne* Bentham (Lamiaceae) was described from elsewhere in Kaua`ula Valley (Wood and Oppenheimer 2008). Additionally, an undescribed taxon in *Tetramolopium* Nees (Asteraceae) is also under study and is known from only Kaua`ula Valley and one additional site, also on West Maui.

When evaluated using the IUCN Red List criteria *Cyanea kauaulaensis* falls into the Critically Endangered (CR) category, a designation reserved for species facing the greatest risk of extinction in the wild, as it fulfills the following criteria: B) Extent of occurrence estimated to be less than 100 km² or area of occupancy estimated to be less than 10 km²; 2c) Continuing decline observed in area, extent and/or quality of habitat; 2c(iv) Continuing decline observed in number of mature individuals; C) Population size estimated to number fewer than 250 mature individuals and C2a(1) no subpopulation estimated to contain more than 50 mature individuals. This species has a known range of less than 100 km², and an area of occupancy of less than 10 km² currently known from three populations, two in Kaua`ula Valley and one in Waikapu Valley, both on West Maui. Furthermore, *Cyanea kauaulaensis* should be considered by the US Fish & Wildlife Service as a Candidate for listing as Endangered under the Endangered Species Act of 1973, and a Recovery Plan written, funded, and implemented.

The Maui Invasive Species Committee (MISC) has been working to control the *Cortaderia* infestation in both valleys on the surrounding, vertical cliffs. The region has escaped the ravages of introduced feral ungulates due to the extremely rugged topography. This new species is a target of the Plant Extinction Prevention Program (PEPP), with efforts made to collect seeds from every individual plant, propagation of nursery stock, restoration outplanting into appropriate habitat, and *ex situ* seed storage. In April of 2009 seeds from 32 of the 45 plants in the north fork subpopulation were collected. The south fork subpopulation was sampled in 2004 and the population seemed stable with twelve plants. It was revisited in October 2010. Only three individuals remained, and the habitat had been significantly degraded by dense stands of *Coffea arabica*. Plants have been successfully grown at Lyon Arboretum, Honolulu, the National Tropical Botanical Garden, Kaua`i, and the Olinda Rare Plant Facility on Maui. In October 2010 84 plants from seeds collected in the north fork were planted in the south fork, in the hopes of augmenting the diminishing population there. In September of 2011, 16 plants from the north fork of Kaua`ula Valley were planted adjacent to the Waikapu population.

#### Specimens examined.

**USA.** Hawaiian Islands. Maui [West Maui]: Lahaina District, AMFAC survey by TNCH, Kauaula Valley, back of valley near streambed, 27 Oct 1989, Perlman & Gon 10841 (F, BISH, PTBG, US); back of Kauaula Valley on west side, 866 m, 2 Dec 2008, Perlman & Oppenheimer 21284 (PTBG, BISH, NY, US), east side fork, 18 Jan. 1995, Perlman et al. 14626 (F, MO, NY, PTBG), 2700 ft. (823m), Perlman et al. 18875 (OSH, PTBG); Kaua`ula Valley, N fork, 3000 ft (914m), 6 Apr 2009 (fr), Oppenheimer et al. H40919 (BISH, PTBG [+ spirit coll.]), Kaua`ula Valley, N fork, 3000 ft (914m), 18 Feb 2009, Oppenheimer & Wood H20928 (PTBG), 2950 ft (899m), 1 July 2009, Oppenheimer & Perlman H70901 (PTBG); North fork headwaters, 2780 ft (847m), 26 Sep 2009, Oppenheimer & Kia 90914 (PTBG), Kauaula Valley, upper south fork below Helu, 2700 ft (823m), 18 Jan 1995, Wood et al. 3940 (PTBG); Wailuku District, Waikapu Valley, north fork, 2400 ft (732m), Jul 2011, Oppenheimer & Bustamente H71103 (BISH, PTBG).

#### Discussion.

Several attempts have been made to divide *Cyanea* into sections (Hillebrand 1888; Rock 1919; Wimmer 1943; St. John 1969, Stone 1967, Lammers 1990), but none successfully arranges the genus into clear-cut phylogenetic entities. Givnish et al. (1995) divide the genus into two distinct clades: one with purple fruits and another with orange fruits. *Cyanea kauaulaensis* belongs to a lineage that previously has not been represented on Maui. Based on its glabrous leaves and similar floral morphology, the new species appears to be most closely related to the rare *Cyanea profuga* C. Forbes of Moloka`i, which was placed by Rock (1919) in his section Genuinae. The latter differs from the new species in having inflorescences with fewer (9–12) flowers, flowers with larger linear-elliptic or oblong calyx lobes 5–9 mm long and 1.2–2 mm wide, longer than the hypanthium, its comparatively longer corolla lobes ¼ to almost ½ as long as the tube, and ellipsoid-cylindrical fruits which also ripen orange. Seedlings of both species grown together at the Olinda Rare Plant Facility are virtually indistinguishable from one another. The islands of Moloka`i, Lana`i, Kaho`olawe, and Maui were once a single, large land mass, referred to as Maui Nui, and their biota shares many components (Price and Elliott-Fisk 2004).

The following couplets can be inserted into the most recent revision of *Cyanea* (Lammers in Wagner et al. 1990) to separate *Cyanea kauaulaensis* from *Cyanea profuga*.

**Table:** 

15(13)	Leaf margins irregularly lobed or cleft; Mo	11 *Cyanea dunbariae*
15	Leaf margins callose-toothed, erose, entire or minutely serrulate	(15’)
15’(15)	Leaf margins entire or minutely serrulate; calyx lobes lanceolate to linear, apex acute to acuminate; M	*Cyanea kauaulaensis*
15’	Leaf margins callose-toothed or erose	(16)
16(15’)	Calyx lobes oblong, apex rounded and apiculate; Mo	38 *Cyanea profuga*
16	Calyx lobes dentiform or triangular, apex acute to acuminate	(17)

## Supplementary Material

XML Treatment for Cyanea kauaulaensis
